# Preparation and Post-Processing of Three-Dimensional Printed Porous Titanium Alloys

**DOI:** 10.3390/ma18081864

**Published:** 2025-04-18

**Authors:** Tairong Li, Mengyu Xu, Jinzhi Yao, Liping Deng, Bingshu Wang

**Affiliations:** 1 College of Materials Science and Engineering, Fuzhou University, Fuzhou 350108, China; l854767407@163.com (T.L.); xumengyu0419@163.com (M.X.); 2 Department of Orthopedics, The Second Affiliated Hospital of Fujian Medical University, Quanzhou 362000, China; 3 School of Mechanical Engineering and Automation, Fuzhou University, Fuzhou 350108, China; ldeng@fzu.edu.cn

**Keywords:** 3D printing, porous titanium alloys, selective laser melting (SLM), chemical polishing, mechanical properties

## Abstract

Ti6Al4V is widely utilized in orthopedic implants due to its excellent mechanical properties, corrosion resistance, and biocompatibility. However, traditional solid titanium implants exhibit an elastic modulus (90–115 GPa) significantly higher than that of human bone (10–30 GPa), leading to stress shielding and implant loosening. To address this, porous titanium alloys have been developed to better match bone elasticity. Additive manufacturing, particularly selective laser melting (SLM), enables precise control over pore size and porosity, thereby tuning mechanical properties. Nevertheless, SLM-produced porous structures often suffer from powder adhesion, which compromises bone integration and patient safety. In this study, bulk Ti6Al4V samples were fabricated via SLM with a fixed laser power of 200 W and varying scanning speeds (800–1400 mm/s). Density measurements and surface defect analysis identified 1200 mm/s as the optimal scanning speed. Cubic unit cell scaffolds with different pore diameters (400, 600, 800 μm) and porosities (60%, 80%) were subsequently designed. Compression tests revealed that scaffolds with a 400 μm pore diameter and 60% porosity exhibited the highest compressive strength (794 MPa) and fracture strain (41.35%). Chemical polishing using a diluted HF-HNO_3_ solution (1:2:97) effectively removed adhered powder without significant structural degradation, with 40 min identified as the optimal polishing duration.

## 1. Introduction

Orthopedic metal implants represent a cornerstone of the biomedical materials industry, with China’s market exceeding 54.8 billion RMB in 2023 [1]. The success of these implants’ hinges on their physical and biological properties, which directly influence surgical outcomes and postoperative patient quality of life [2]. Titanium alloys, particularly Ti6Al4V, have emerged as a gold standard for bone repair and fixation due to their exceptional biocompatibility, corrosion resistance, and favorable mechanical properties. Abdel-Hady [3] pointed out that, although titanium alloys have drawn attention because of potential health risks, they remain essential materials for bone repair and fixation. Titanium alloys possess excellent biocompatibility, corrosion resistance, and appropriate mechanical properties. Geetha [4] focused on titanium-based biomaterials and elucidated that titanium alloys, especially Ti6Al4V, exhibit remarkable advantages within the realm of orthopedic implants. This can be attributed to the excellent biocompatibility, strong corrosion resistance, and favorable mechanical properties of titanium alloys. Upon implantation, a dense TiO_2_ oxide layer forms on the alloy surface, mitigating immune rejection and enhancing osseointegration [5]. Despite these advantages, conventional solid titanium implants exhibit a critical limitation: their elastic modulus (90–115 GPa) starkly exceeds that of human cortical bone (10–30 GPa), leading to stress shielding effects that compromise long-term implant stability [6].

Porous titanium alloys offer a transformative solution by mimicking the trabecular architecture of natural bone. By tailoring porosity and pore diameter, the elastic modulus can be reduced to ~20 GPa, effectively aligning with bone mechanics. These interconnected porous networks also facilitate nutrient diffusion and osteoblast proliferation, accelerating bone ingrowth and mechanical interlocking. Gai [7] found that, in the open-cell structured Ti6Al4V alloy prepared by electron beam melting, the interconnected porous network significantly affects its electrochemical properties. Additionally, this porous structure facilitates the diffusion of nutrients and the proliferation of osteoblasts, which accelerates bone ingrowth and enhances mechanical locking effects. Rodriguez [8] prepared a porous titanium structure using the powder metallurgy method combined with a pore-forming agent. This structure features an interconnected porous network that facilitates the diffusion of nutrients and the proliferation of osteoblasts, thereby accelerating bone ingrowth and enhancing mechanical locking. Furthermore, it effectively avoids the stress shielding effect, demonstrating exceptional performance in orthopedic implant applications. Previous studies have demonstrated that porous titanium alloys are capable of promoting osteoblast adhesion and proliferation, with their porous architecture significantly enhancing new bone ingrowth. Sarraf [9] indicated that titanium alloys are extensively utilized within the biomedical field. Their porous architecture has the capacity to facilitate the adhesion and proliferation of osteoblasts, as well as augment new bone ingrowth. Moreover, this particular property can be further refined by means of diverse surface modification approaches and advanced manufacturing technologies. Yuan [10] aimed to solve the problems of infection and bone integration of titanium implants. Using various surface design strategies, the implants can be endowed with antibacterial and bone integration-promoting properties. Among them, porous titanium alloys can promote the adhesion and proliferation of osteoblasts and accelerate new bone ingrowth while achieving effective antibacterial effects. Gulati [11] pointed out that when nanoengineered titanium implants are used for craniofacial treatment, their porous structure helps to promote the adhesion and proliferation of osteoblasts and enhance new bone ingrowth. These porous structures also play an important role in bone treatment, soft tissue integration, and other aspects. The bond strength between bone and porous titanium alloy is notably stronger compared to solid titanium alloys. Moreover, extensive literature [12] supports the notion that the porous structure of these materials is advantageous for both macroscopic and microscopic aspects of bone repair and reconstruction.

In recent years, numerous studies have shown that the titanium alloy Ti6Al4V may have adverse health effects. Aluminum and vanadium may exhibit cytotoxic effects, potentially leading to DNA damage and other complications [3]. Aluminum ions (Al^3+^) could accumulate in the central nervous system through the blood–brain barrier. Although the amount of aluminum released from the titanium alloy is extremely low (<1 μg/L/year), long-term accumulation might pose risks to sensitive individuals. Vanadium ions (V^5+^) have been demonstrated to be genotoxic, capable of inducing oxidative stress and DNA damage. Despite these concerns, Ti6Al4V remains the “gold standard” for orthopedic and dental implants. This is attributed to the excellent biocompatibility of Ti6Al4V, which enables it to integrate seamlessly with human tissues and minimize immune rejection. Its mechanical properties closely mimic those of natural bone, allowing it to effectively bear physiological loads and reduce the stress-shielding effect. Additionally, it exhibits superior corrosion resistance in the human physiological environment, ensuring long-term stability. The alloy’s versatile processability facilitates the manufacturing of complex medical devices, while stringent quality control standards further mitigate potential risks. Owing to these combined strengths, Ti6Al4V retains its irreplaceable status in biomedical titanium applications.

Traditional methods for preparing porous titanium alloys, such as the sintering method [13], the foaming method [14], and powder metallurgy [15], often result in uncontrollable pore diameters and porosities. Consequently, the mechanical properties of the resulting materials are less than ideal, failing to meet the stringent performance criteria required for bone tissue engineering scaffolds. However, the advent and advancement of additive manufacturing technology have demonstrated significant potential in this field. Cheng [16] utilized laser sintering to fabricate porous titanium alloys with varying porosities. The elastic modulus of these alloys ranged from 2.5 to 3.6 GPa (with porosity levels between 15% and 70%), although the pore sizes across the scaffolds were inconsistent. Crovace [17] employed electron beam melting to produce porous titanium alloy scaffolds, which exhibited excellent osteoconductive properties and facilitated bone ingrowth according to in vivo testing results. Fousova [18] leveraged selective laser melting (SLM) technology to create porous titanium alloy scaffolds featuring three distinct gradient porosities. Notably, a scaffold with 61% porosity achieved an elastic modulus of 30.5 GPa, closely matching that of human cortical bone and effectively mitigating stress shielding effects. Furthermore, cell culture tests confirmed the biocompatibility of the SLM-fabricated titanium alloy. Additionally, Onal [19] explored the use of SLM technology to develop porous titanium alloy scaffolds with body-centered cubic (BCC) lattice structures, achieving continuously graded porosity by adjusting the strut diameters. This research highlighted the fact that the fabricated scaffolds possessed mechanical properties analogous to those of natural bone and that the larger pore sizes promoted higher proliferation rates of osteoblasts within the scaffolds.

For porous titanium alloy scaffolds fabricated via selective laser melting (SLM), the Gaussian distribution of laser energy leads to the localized melting of powder particles near the struts during the formation process due to heat exposure. Upon solidification, these particles adhere to the struts. A considerable number of partially melted powder particles remain attached to the formed porous structure. Yang [6] pointed out that, during the preparation of porous titanium, in processes such as selective laser melting, a large number of partially melted powder particles adhere to the formed porous structure. This phenomenon will affect the pore structure, surface quality, and mechanical properties of porous titanium. Luo [20] prepared porous Ti6Al4V using in situ double scanning during laser additive manufacturing. The double scanning increased the laser energy, causing a large number of partially melted powder particles to adhere to the formed porous structure, which affected the porosity, surface quality, and dimensional accuracy of the samples. These particles are only weakly connected to the struts over relatively small contact areas. Consequently, when implanted into the human body, such particles are susceptible to detachment by bodily fluid corrosion, potentially entering the body and adversely affecting bone growth and overall health [21]. Thus, effectively removing these adherent powder particles is crucial. Research has indicated that chemical polishing can successfully eliminate these particles while enhancing the osteogenic performance of scaffolds. Wysocki [22] fabricated Ti6Al4V scaffolds with varying pore diameters using SLM and subsequently subjected them to chemical polishing. The findings revealed that polishing with a single HF solution caused significant damage to the scaffolds, whereas an HF-HNO_3_ solution could thoroughly remove residual powder while preserving scaffold integrity more effectively. However, employing highly concentrated acidic solutions for polishing risks damaging the struts during the powder removal process. Therefore, this study proposes utilizing a less concentrated acidic solution for polishing while extending the polishing duration. This approach aims to achieve effective powder particle removal while minimizing adverse effects on the structural integrity of the struts. This strategy ensures enhanced biocompatibility and mechanical stability of the scaffolds, making them more suitable for medical applications.

The surface state of medical porous titanium alloys plays a pivotal role in determining their performance, exerting profound multi-dimensional impacts. Surface roughness, pore structure, and chemical composition directly govern the adhesion, proliferation, and differentiation of bone cells. Surface wettability, for instance, regulates the spreading behavior of synovial fluid or tissue fluid on the material surface, thereby significantly influencing lubrication performance and anti-biofouling capabilities. Bartkowiak [23] discovered that additively manufactured surfaces featuring specific textures hold the potential to modulate surface wettability. This modulation, in turn, can effectively change the spreadability of synovial fluid or other human body fluids on the implant surface. Peta [24] demonstrated that electrical discharge machining (EDM) parameters exert significant influence over the surface microstructural architecture of Ti6Al4V alloy, thereby dictating the wettability behavior of biomimetic synovial fluid analogs with varying viscosities. The authors found that elevated discharge energy induces increased surface roughness and augmented contact angles, impeding the spreading dynamics of high-viscosity synovial fluid mimics, whereas microstructural features at specific length scales emerge as critical determinants in governing wetting phenomena. The surface oxide layer or functional coating serves to enhance the long-term stability of porous titanium alloys within the body fluid environment, minimizing the release of metal ions. The surface treatment further optimizes the stress distribution within the porous structure. By reducing the stress-shielding effect, it substantially extends the lifespan of the implant. Additionally, the surface state also impacts physical and chemical properties such as the surface energy of porous titanium alloys. These combined characteristics work in concert to comprehensively enhance the overall performance of porous titanium alloys in the medical field. This enhanced performance thereby lays a solid foundation for their more extensive clinical applications.

In this study, we initially maintained constant laser power while varying the scanning speed. By conducting density tests and surface defect observations, we investigated the impact of scanning speed on bulk samples to determine the optimal printing parameters. Subsequently, we designed six cubic unit cell porous titanium alloy scaffolds, each with distinct pore diameters and porosities. Mechanical property testing of these samples enabled us to identify the ideal scaffold structure. To address the issue of powder adhesion on scaffold surfaces, we employed chemical polishing for surface treatment of the porous titanium alloy scaffolds. This process was crucial in ensuring the scaffolds’ surfaces were free from residual powder particles, thereby enhancing their biocompatibility and mechanical performance. Through this systematic approach, we were able to optimize both the fabrication parameters and post-processing methods, resulting in superior scaffold designs tailored for bone tissue engineering applications.

## 2. Materials and Methods

Ti6Al4V powder with a particle size ranging from 15 μm to 53 μm was sourced from Bright Laser Technologies (Xi’an, China). Selective laser melting (SLM) was utilized for the printing process, employing the BLT-A160 machine (Bright Laser Technologies, Xi’an, China). For the fabrication of titanium alloy bulk samples, the laser power was fixed at 200 W, while the scanning speed was varied between 800 mm/s, 1000 mm/s, 1200 mm/s, and 1400 mm/s. The scanning interval was set at 0.09 mm, and the layer thickness was 0.03 mm. During the SLM process, a significant temperature gradient is generated, which can lead to the development of substantial thermal stress within the formed specimens. As the number of printed layers increases, thermal stress accumulates, potentially causing warping or cracking of the specimens [25]. Research has indicated that continuous exposure scanning strategies are prone to the accumulation of thermal stress, resulting in the warping and cracking of large-sized specimens during the forming process [26]. In contrast, a 67° alternating scanning strategy significantly mitigates thermal stress accumulation. Consequently, the 67° alternating scanning strategy was adopted for this bulk-forming experiment. Table 1 provides a summary of the SLM printing parameters used.

**Figure 1:**
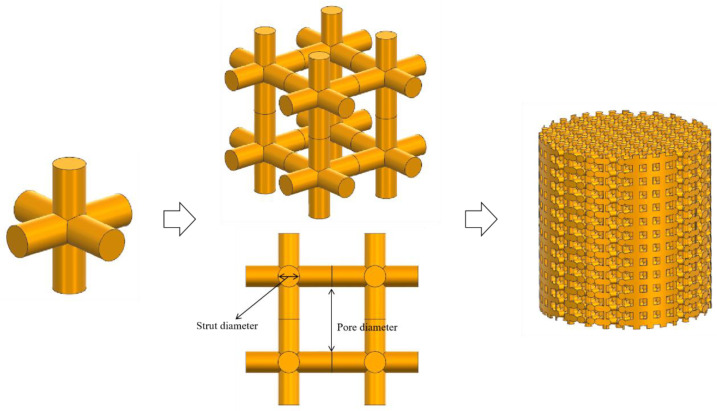
Modeling process of cube unit cell porous titanium alloy scaffolds composed of cube structural units.

We utilized NX2312.1700 software (Siemens Digital Industries Software, 2023, Munich, Germany) to construct the scaffold model. The detailed modeling process for the porous titanium alloy scaffold is illustrated in Figure 1. Initially, we designed the structural unit of the cubic unit cell for the porous titanium alloy scaffold. Subsequently, multiple unit cells were connected following a cubic arrangement, and a Boolean intersection operation was performed between these units and a cylinder. This process resulted in a porous titanium alloy scaffold test sample with a diameter of 10 mm and a height of 10 mm, comprising several small cubic structural units. The pore diameter and porosity of the scaffold were controlled by adjusting the diameter and height of the struts. The specific dimensions used in the design are summarized in Table 2.

Archimedes’ principle of water displacement [27] was employed to determine the relative density of the bulk samples. The procedure was as follows: First, the weight of the samples was measured in air, denoted as W1, using an electronic balance. Next, the samples were suspended by a thin thread and fully submerged in water, and their weight while submerged, W2, was recorded. From these measurements, the actual density of the samples was calculated. Finally, the relative density was determined by dividing the actual density by the theoretical density of the Ti6Al4V alloy. The formula for calculating the relative density is given below:

(1) $$P=\frac{\rho }{\rho 0}\times 100\%=\frac{W1\times \rho H2O}{(W1-W2)\times \rho 0}\times 100\%$$

We ground the samples formed by selective laser melting (SLM) on sandpaper. Subsequently, the ground samples were polished on a polishing machine, and a silica polishing agent with a particle size of w0.04 was used for the polishing. Then, we utilized a metallographic microscope and a thermal field emission scanning electron microscope (Carl Zeiss, Oberkochen, Germany) to observe the surface defects of the bulk samples.

A thermal field emission scanning electron microscope was used to scan the pore structure of the porous titanium alloy scaffolds. Then, measurements were carried out on the scanned images using Image software (ImageJ 1.54g). Measurements were taken 50 times at different locations on each scaffold, and the average value was calculated [28]. The mass method was adopted to determine the porosity of the porous titanium alloy. The experimental equipment used was an electronic balance. Before the measurement, we first performed ultrasonic cleaning on the samples to fully remove the powder remaining in the pores. After that, the samples were completely dried. Then, the mass, M, of the samples was weighed using an electronic balance, and the apparent volume, V0, of the porous titanium alloy was measured and calculated with a vernier caliper. The calculation formula for the porosity is:

(2) $$P=\frac{V0-V}{V}=1-\frac{M}{V0\times \rho }$$

According to the ISO13314:2011 standard [29], a CMT5504 universal testing machine was used to conduct a compression test along the forming direction of the sample. The size of the compressed sample was set to a diameter of 10 mm and a height of 15 mm, and the compression rate was set to 0.5 mm/min.

Chemical polishing was employed to eliminate adhered powder particles within porous Ti6Al4V scaffolds fabricated via selective laser melting (SLM). The scaffolds measured 10 mm in diameter and 10 mm in height. A polishing solution with a composition ratio of HF:HNO_3_:H_2_O = 1:2:97 was used, and the samples underwent polishing for durations of 10, 20, 30, and 40 min. To ensure uniform polishing, the samples were placed in a 100 mL beaker to which 25 mL of the polishing solution was added. The beaker containing the samples was then placed in an ultrasonic cleaner for chemical polishing. Upon completion of the polishing process, the samples were subjected to ultrasonic cleaning in anhydrous ethanol for 20 min, followed by a 10 min ultrasonic rinse in deionized water to thoroughly remove any residual powder particles. Finally, the samples were dried to prepare them for further analysis or use.

## 3. Results

### 3.1. Results of Bulk Samples Under Different Scanning Speeds

**Figure 2:**
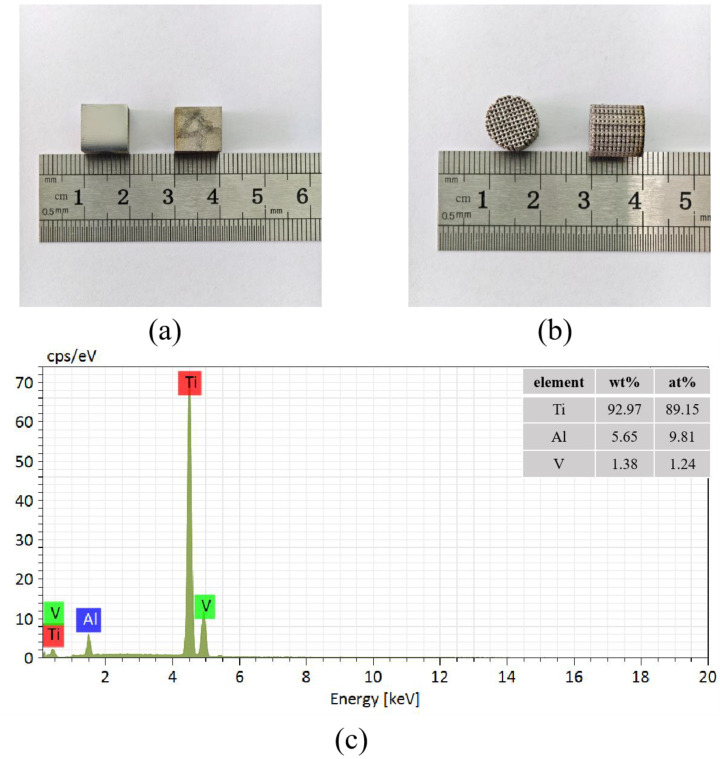
(**a**) Cubic block sample; (**b**) Cylindrical porous Ti6Al4V scaffold sample; (**c**) The chemical composition of the fabricated samples detected by EDS.

Figure 2a,b illustrate two types of samples manufactured using selective laser melting (SLM) with Ti6Al4V powder. The first type is a cubic block, with all dimensions—length, width, and height—measuring 10 mm. This sample was utilized for density testing and surface defect observation. The second type is a cylindrical porous titanium alloy sample, featuring a diameter of 10 mm and a height of 10 mm, designed for compression property testing and chemical polishing experiments.

**Figure 3:**
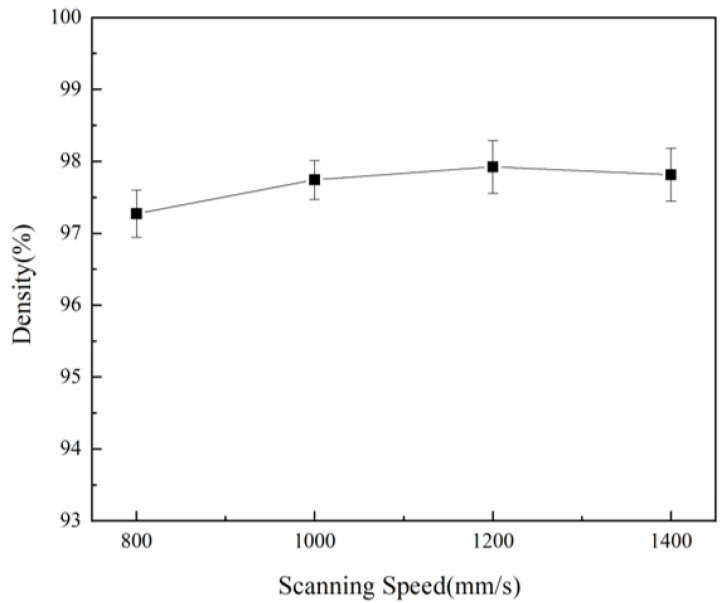
The density of bulk samples formed at different scanning speeds.

Figure 3 illustrates the density curves of bulk samples printed at various scanning speeds. As the scanning speed increased, the sample density initially rose before declining. At a scanning speed of 800 mm/s, the samples exhibited their lowest density, measuring 97.27%. With an increase in scanning speed, the sample density improved, reaching its peak value of 97.93% at 1200 mm/s. However, as the scanning speed continued to increase, the sample density decreased; at 1400 mm/s, the density fell to 97.81%.

**Figure 4:**
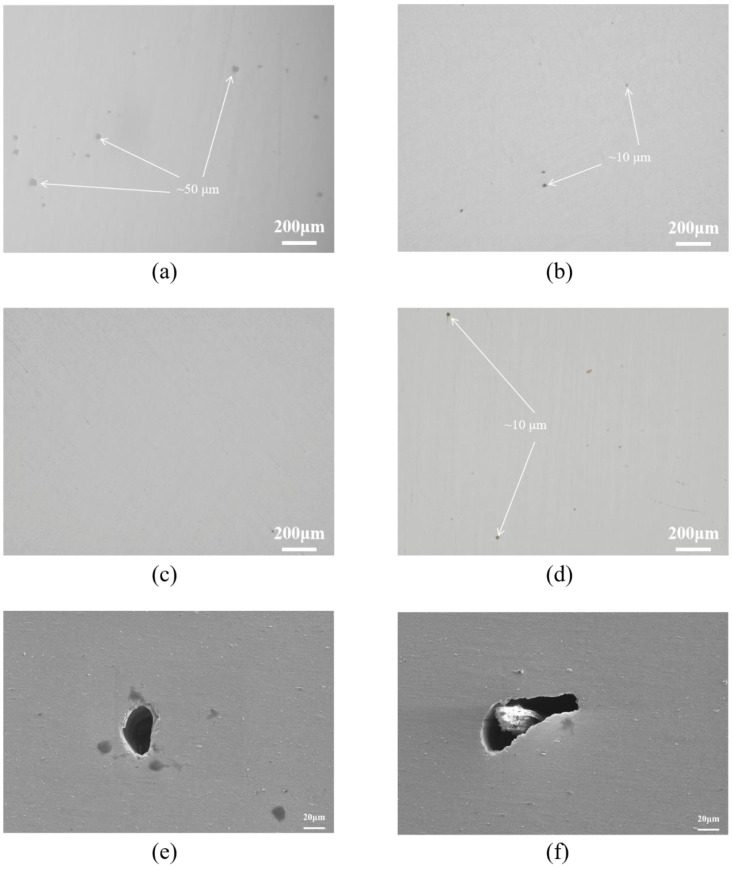
(**a**–**d**) OM images of the XOY plane of bulk samples formed at different scanning speeds. (**a**) v = 800 mm/s; (**b**) v = 1000 mm/s; (**c**) v = 1200 mm/s; (**d**) v = 1400 mm/s; (**e**,**f**) Defect morphologies on the XOY plane of bulk samples at different scanning speeds. (**e**) v = 800 mm/s; (**f**) v = 1400 mm/s.

Figure 4a–d present metallographic microscope images of bulk samples fabricated at different scanning speeds. These figures reveal the presence of defects varying in shape and size across the samples. For samples printed at a scanning speed of 800 mm/s, a relatively high number of defects were observed. These included numerous small, circular pores (~10 μm in diameter) as well as larger voids (~50 μm in diameter). At a scanning speed of 1000 mm/s, the large defects disappeared, and the size of the remaining circular pores decreased. When the scanning speed was increased to 1200 mm/s, the samples exhibited significantly fewer and smaller defects. However, increasing the scanning speed to 1400 mm/s resulted in the reappearance of relatively large defects and an increase in their number compared to those observed at 1200 mm/s.

To further investigate the impact of scanning speed on defect formation, we employed a thermal field emission scanning electron microscope to examine the defect morphology more closely, as shown in Figure 4e,f. During the selective laser melting (SLM) process, a slower scanning speed leads to a higher volumetric energy density, which can cause keyhole formation within the melt pool [30], as illustrated in Figure 4e. Conversely, a higher scanning speed results in a lower volumetric energy density, leading to incomplete fusion defects within the samples, as depicted in Figure 4f.

### 3.2. The Micro-Pore Structure of the Scaffold

**Figure 5:**
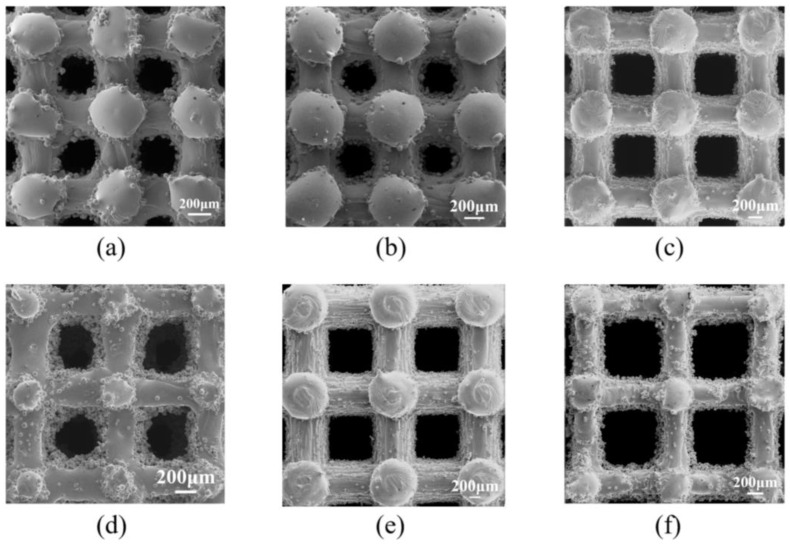
(**a**–**f**) Morphology diagrams of scaffolds with different pore diameters and porosities. (**a**) P400-60; (**b**) P400-80; (**c**) P600-60; (**d**) P600-80; (**e**) P800-60; (**f**) P800-80.

The porous scaffolds were examined using a scanning electron microscope (SEM). Figure 5 illustrates the strut morphologies of porous scaffolds with varying porosities. The struts of the porous Ti6Al4V scaffolds fabricated via selective laser melting (SLM) exhibited continuity and were free from macroscopic flaws such as fractures or misalignments, with pore geometries remaining well-defined. Notably, a significant number of un-melted powder particles were observed adhering both within the scaffold structures and in the vicinity of the struts [31].

The actual pore diameter sizes of selective laser melting (SLM) titanium alloy scaffolds, as measured from SEM images, are summarized in Table 3. Both the actual pore diameters and porosities of the printed scaffolds were found to be smaller than the designed values. Furthermore, it was observed that the smaller the pore diameter, the greater the discrepancy between the actual and designed values. Conversely, the strut diameters of the scaffolds were all larger than the designed dimensions. Additionally, the smaller the designed strut size, the higher the likelihood of un-melted Ti6Al4V powder adhering around the pores. These two factors, reduced pore diameters and increased strut sizes due to adhered powder, are the primary reasons why the actual pore diameters and porosities of the scaffolds were lower than the designed specifications.

**Figure 6:**
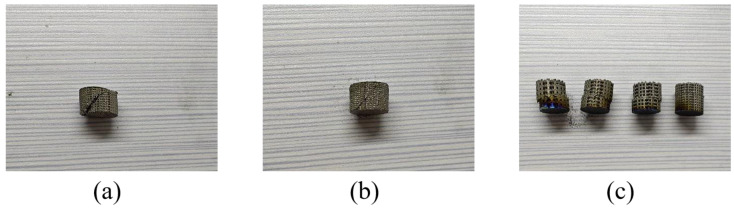
(**a**–**c**) Macroscopic fractured photos of the scaffolds. (**a**) P400-60; (**b**) P400-80; (**c**) The other scaffolds.

To investigate the compressive properties of porous Ti6Al4V, compression tests were conducted on titanium alloy samples fabricated via selective laser melting (SLM). As depicted in Figure 6a–c, macroscopic fracture images reveal that samples P400-60 and P400-80 fractured at an angle of approximately 45° relative to the loading direction following compressive deformation. In contrast, for samples P600-60, P600-80, P800-60, and P800-80, after a slight initial deformation, all scaffolds within a specific internal layer fractured, leading to a horizontal dislocation between the fractured parts and the remaining portions of the samples.

**Figure 7:**
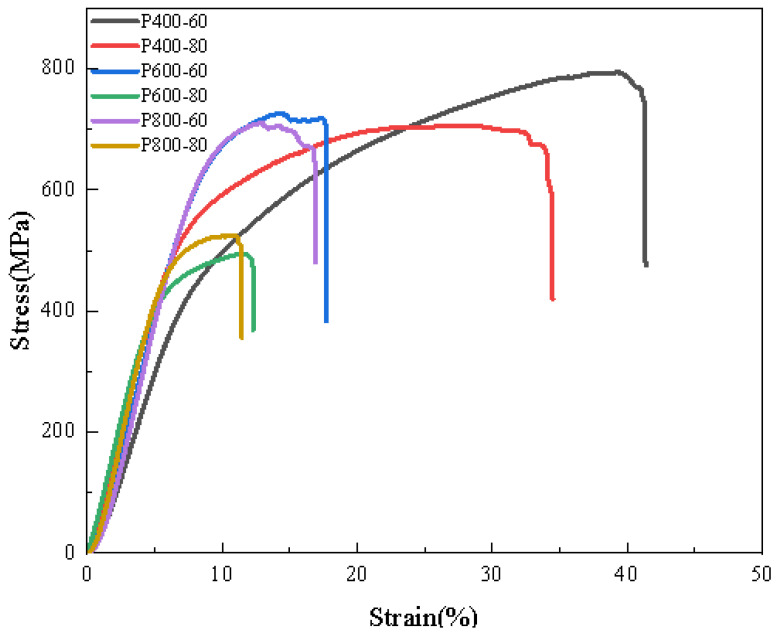
The curves are the compressive stress–strain curves of porous titanium alloy scaffolds.

The compressive stress–strain curves of porous Ti6Al4V are presented in Figure 7, with Table 4 detailing the specific compressive properties of the porous titanium alloy scaffolds. Once implanted into the human body, porous scaffolds are subjected to complex stresses [32]. For bone scaffolds that must bear loads, it is crucial they exhibit high strength and good stability. Premature fractures indicate scaffold failure; thus, these structures must also demonstrate excellent plastic deformation capabilities to avoid breaking under pressure [33]. From the stress–strain diagrams of the porous Ti6Al4V, it is evident that sample P400-60 achieved a maximum compressive strength of 794 MPa and a maximum fracture strain of 41.35%. In contrast, the stress–strain curves for P600-60, P600-80, P800-60, and P800-80 indicated that these samples experienced relatively minimal plastic deformation before fracturing. Consequently, P400-60 demonstrated superior suitability as an orthopedic implant due to its enhanced mechanical properties and greater capacity for plastic deformation.

### 3.3. Results of the Influence of Chemical Polishing Time on the Scaffolds

Following chemical polishing, the adhered powder within the pores of the porous scaffolds was progressively eliminated. The pore shapes became more regular, and the strut widths exhibited greater uniformity. As shown in Figure 7, after 10 and 20 min of chemical polishing, most of the powder on the struts had been removed, although a considerable number of powder particles remained inside the pores. When the polishing duration was extended to 30 min, the surface powder particles were entirely removed, leaving only a small number of particles inside the pores. After 40 min of polishing, nearly all powder particles within the porous scaffolds were removed, with only a few burs remaining inside the pores.

Table 5 details the changes in pore diameter, porosity, and strut diameter of the porous scaffolds following various polishing durations. As the polishing time increased, both the pore diameter and porosity of the scaffolds gradually increased, while the strut diameter progressively decreased. It is important to note that, although the strut diameter decreased to varying extents, no significant macroscopic defects, such as drastic reductions in strut diameter or localized fractures, were observed.

**Figure 8:**
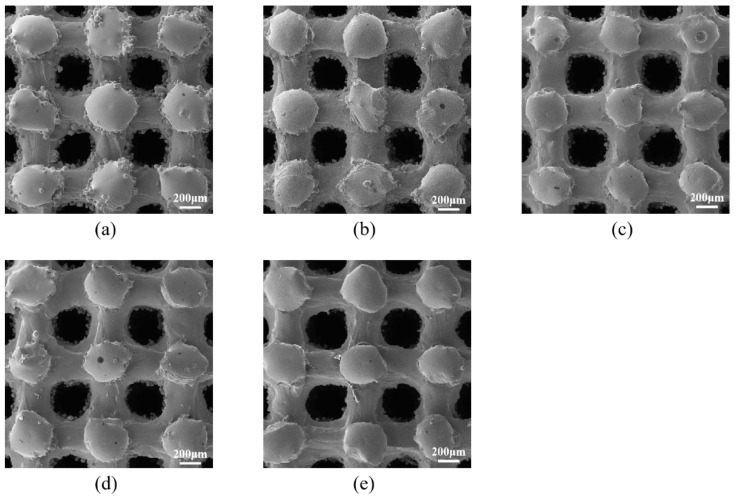
Surface morphology diagrams of P400-60 after chemical polishing for different durations. (**a**) P400-60; (**b**) P400-60-10; (**c**) P400-60-20; (**d**) P400-60-30; (**e**) P400-60-40.

**Figure 9:**
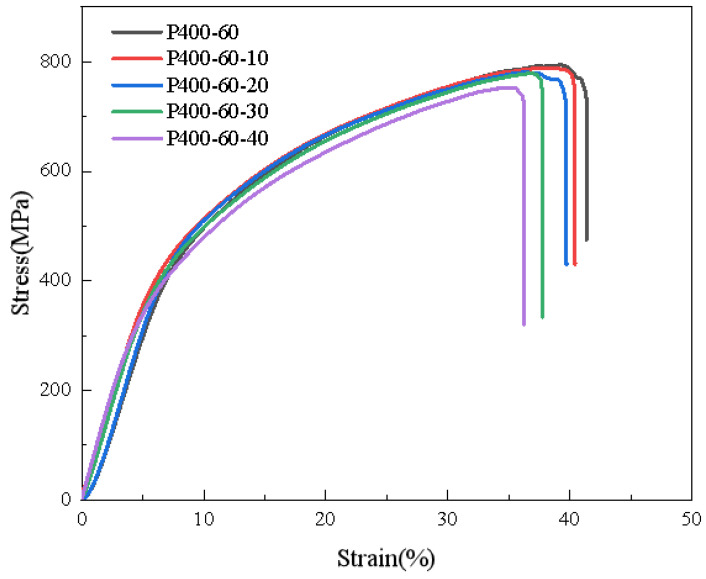
The compressive stress–strain curves of P400-60 under different polishing times.

Given that chemical polishing reagents simultaneously remove adhered powder particles and slightly corrode the struts, further investigation into the impact of chemical polishing on porous titanium alloy scaffolds was conducted through compression performance tests at various polishing durations. Figure 8 illustrates the stress–strain curves of the scaffolds under different polishing times, while Table 6 provides detailed values of the compressive properties for these scaffolds. As shown in Figure 9, it is evident that polishing time had a relatively minor impact on the strength of the porous titanium alloy scaffolds. With increasing polishing time, both the compressive strength and fracture strain of the scaffolds gradually decreased. Specifically, after 40 min of chemical polishing, the compressive strength of P400-60 decreased from 794 MPa to 753 MPa, and its fracture strain dropped from 41.35% to 36.20%. It is important to note that, despite this reduction, the scaffolds maintained good stability during compression testing, with no sudden fractures observed in local struts. This indicates that, although chemical polishing effectively removed adhered powder particles, it did not significantly degrade the mechanical properties of the porous scaffolds. Based on the results of the compression performance tests, even after 40 min of chemical polishing P400-60 still exhibited relatively high strength and stability. Therefore, a polishing duration of 40 min appears to be an appropriate choice, balancing the removal of adhered powder particles with minimal adverse effects on mechanical performance.

After pickling, the fluorine element may exist on the scaffold in two forms: (1) Free F^−^ (physical adsorption): Residual fluoride ions (F^−^) that are not thoroughly cleaned after pickling may be adsorbed on the surface or inside of the pores. (2) Chemically bonded F (Ti-F bonds): HF reacts with the titanium matrix to form titanium fluorides (such as TiF_3_ and TiF_4_), which are embedded in the surface oxide layer or the inner wall of the pores. The potential positive effects of the fluorine element are as follows: (1) F^−^ inhibits biofilm formation by interfering with bacterial metabolic enzymes (such as enolase), reducing the risk of infection. Thus, the antibacterial performance of the scaffold is improved. (2) A trace amount of F can promote osseointegration. The negative impacts and risks of the fluorine element include cytotoxicity, inflammatory response, deterioration of mechanical properties, and long-term corrosion risks. Therefore, it is very necessary to detect the fluorine element.

**Figure 10:**
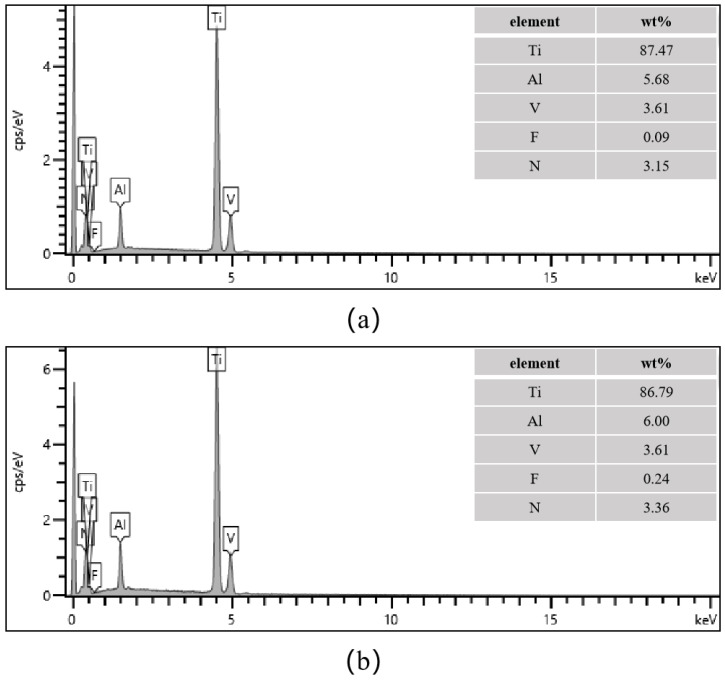
The chemical composition of the fabricated samples detected by EDS. (**a**) before chemical polishing; (**b**) after chemical polishing.

EDS analysis of the samples before and after polishing reveals that the content of the fluorine (F) element increases from 0.09% to 0.24%, as shown in Figure 10. Therefore, XPS is needed to determine whether the fluorine element exists as free fluoride ions or in the form of Ti-F bonds. We fully recognize the importance of XPS analysis in determining the chemical state of the fluorine element and its biological implications. In the subsequent stage of this study, we will conduct high-resolution XPS analysis to distinguish the chemical states of the fluorine element and correlate them with the in vitro cytotoxicity results.

**Figure 11:**
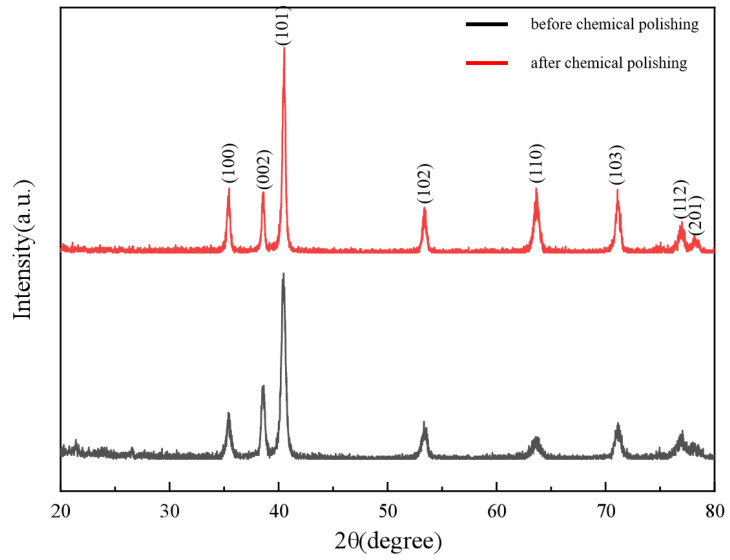
XRD tests of the samples before and after chemical polishing.

XRD analysis revealed consistent diffraction peak positions between pre- and post-polishing patterns, as shown in Figure 11, confirming no alteration in the crystallographic structure of the porous titanium alloy. Variations in peak intensities were attributed to changes in surface grain orientation, residual stress, or topography induced by polishing. Chemical polishing did not introduce extraneous phases, as indicated by the absence of impurity peaks and unaltered peak width/sharpness, while preserving the dominant titanium alloy phase and crystallographic integrity.

**Figure 12:**
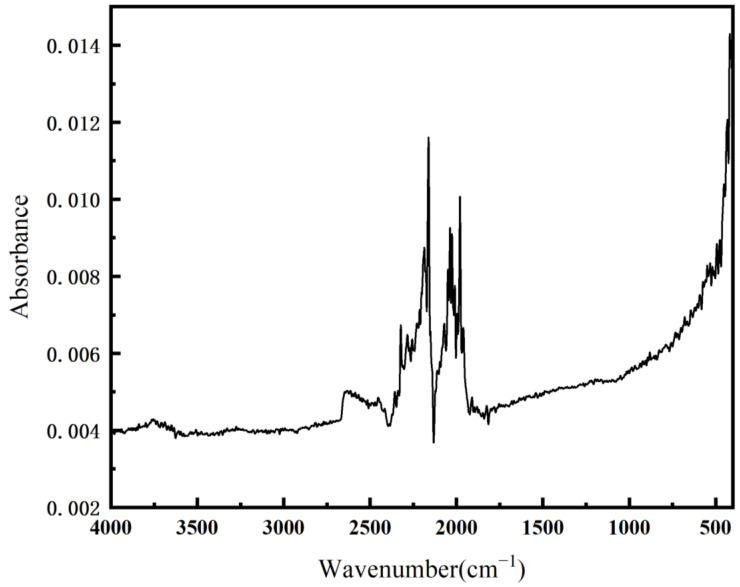
FTIR test of the treated Ti6Al4V sample.

In order to ensure that the treated sample is free from organic contamination, Fourier transform infrared spectroscopy (FTIR) analysis was conducted on the titanium alloy sample, as depicted in Figure 12. Over the wavenumber range spanning from 4000 to 400 cm^−1^, no characteristic absorption peaks corresponding to typical organic functional groups were detected. In detail, within the 3000–3500 cm^−1^ interval, no absorption features associated with the stretching vibrations of hydroxyl (O-H) or amino (N-H) groups were present. In the 2800–3000 cm^−1^ range, no stretching vibration peaks of C-H bonds in alkane moieties were discernible. In the 1600–1800 cm^−1^ region, no absorption signals attributable to carbonyl (C=O) groups and carbon-carbon double bonds (C=C) were observed. Moreover, in the 1000–1300 cm^−1^ range, no peaks related to the stretching vibrations of C-O bonds, such as those in ether linkages and ester functional groups, were manifested. This result indicates that there is no residual organic contamination on the surface of the treated titanium alloy. The FTIR results confirm that the treatment procedure effectively eliminates surface-bound organic substances, meeting the experimental requirements for removing organic contamination from the sample surface and rendering the sample suitable for use as an implant.

## 4. Discussion

The scanning speed during selective laser melting (SLM) significantly influences the surface defect morphology of fabricated samples [34]. Defect morphologies of bulk samples printed at scanning speeds of 800 mm/s and 1400 mm/s were examined using scanning electron microscopy, as depicted in Figure 4e,f. Keyhole defects were observed in the sample printed at 800 mm/s, whereas incomplete fusion defects were noted in the sample printed at 1400 mm/s. This variation is attributed to the effect of scanning speed on laser energy input into the powder [35]. At lower scanning speeds, the volumetric energy density becomes excessively high, leading to an increased width and depth of the melt pool and a rise in melt pool temperature. Consequently, significant evaporation of liquid metal occurs, resulting in pore defects upon solidification. Conversely, at higher scanning speeds, insufficient energy is delivered to the powder, causing incomplete melting. These un-melted particles disrupt the powder spreading for subsequent layers, potentially remaining within the matrix if not melted during later scans, thereby forming cavities around them and impairing layer bonding, which reduces overall density.

Statistical analysis comparing the actual pore diameter, porosity, and strut diameter of the scaffolds with their designed values revealed that both the actual pore diameter and porosity were smaller than intended [36]. During SLM, the laser melts metal powder to create a melt pool, generating considerable heat. Since printing is performed layer by layer, each new layer transfers residual heat to previously printed sections, causing re-heating and thermal expansion of the solidified metal structure. This heat accumulation leads to strut diameters larger than the designed dimensions. Additionally, the issue of powder adhesion to struts further contributes to the scaffold’s actual porosity being less than the designed value [32].

Chemical polishing was applied to P400-60 to investigate its impact on the scaffold over different durations. The results indicated that even a low concentration polishing solution could effectively remove partially melted powder adhered to the surface of porous Ti6Al4V scaffolds. Compression tests confirmed that the polished scaffolds retained high strength, demonstrating the effectiveness of chemical polishing in enhancing scaffold quality without compromising mechanical integrity.

## 5. Conclusions

In this study, the influence of scanning speed on the quality of printed samples was examined. It was observed that both relatively low and high scanning speeds resulted in an increased number of defects within the samples. To address the challenge of the high elastic modulus associated with titanium alloy implants post-implantation, porous titanium alloy scaffolds with varying pore sizes and porosities were manufactured using selective laser melting (SLM) technology. It was found that introducing porosity into the titanium alloy could effectively lower its compressive modulus, and by adjusting the pore size and porosity, the mechanical properties of the scaffolds could be tailored accordingly. Moreover, to resolve the issue of incompletely melted powder particles adhering extensively inside the printed scaffolds, chemical polishing was employed as a remediation technique. Research has demonstrated that utilizing a low-concentration polishing solution not only effectively removes powder adhesion on the scaffold surface but also minimally impacts the mechanical properties of the scaffolds.

## Figures and Tables

**Table 1:** Summary of SLM printing parameters.

Laser Beam Power (W)	Scan Speed (mm/s)	Scanning Spacing (mm)	Layer Thickness (mm)
200	800	0.09	0.03
	1000		
	1200		
	1400		

**Table 2:** Specifications of porous titanium alloy scaffolds.

Scaffold Name	Pore Diameter (μm)	Porosity (%)	Strut Diameter (μm)
P400-60	400	60	410
P400-80	400	80	198
P600-60	600	60	616
P600-80	600	80	296
P800-60	800	60	820
P800-80	800	80	394

**Table 3:** Statistics on the pore structure parameters of porous titanium alloy scaffolds.

Scaffold Name	Pore Diameter (μm)		Porosity (%)		Strut Diameter (μm)	
	Design Value	Actual Value	Design Value	Actual Value	Design Value	Actual Value
P400-60	400	348 ± 10	60	43.50 ± 0.14	305	362 ± 18
P400-80	400	322 ± 11	80	52.76 ± 0.07	198	349 ± 26
P600-60	600	584 ± 26	60	56.97 ± 0.46	616	657 ± 25
P600-80	600	550 ± 13	80	65.51 ± 0.25	296	343 ± 21
P800-60	800	782 ± 13	60	57.27 ± 0.18	820	849 ± 17
P800-80	800	760 ± 16	80	74.70 ± 0.42	394	415 ± 24

**Table 4:** Compressive properties of porous titanium alloy scaffolds.

Scaffold Name	Elastic Modulus (GPa)	Yield Strength (MPa)	Compressive Strength (MPa)	Fracture Strain (%)
P400-60	7.1	365	794	41.3
P400-80	7.9	474	704	34.5
P600-60	7.7	453	717	17.3
P600-80	6.8	351	494	11.5
P800-60	8.3	523	705	14.1
P800-80	10.1	335	524	11.0

**Table 5:** The influence of chemical polishing on pore diameter, porosity, and strut diameter.

Scaffold Name	Pore Diameter (μm)	Porosity (%)	Strut Diameter (μm)
P400-60	345 ± 19	43.50	362 ± 18
P400-60-10	356 ± 20	44.39	355 ± 16
P400-60-20	367 ± 17	44.96	347 ± 19
P400-60-30	374 ± 16	45.91	340 ± 15
P400-60-40	387 ± 17	46.33	334 ± 15

**Table 6:** Compressive properties of porous titanium alloy scaffolds under different polishing times.

Scaffold Name	Elastic Modulus (GPa)	Yield Strength (MPa)	Compressive Strength (MPa)	Fracture Strain (%)
P400-60	7.1	365	794	41.35
P400-60-10	7.7	375	789	40.39
P400-60-20	7.3	388	781	39.70
P400-60-30	7.5	362	779	37.72
P400-60-40	7.8	323	753	36.20

## Data Availability

The original contributions presented in the study are included in the article, further inquiries can be directed to the corresponding author.
